# Serum Proteome Analysis in Patients with Rheumatoid Arthritis Receiving Therapy with Tocilizumab: An Anti-Interleukin-6 Receptor Antibody

**DOI:** 10.1155/2013/607137

**Published:** 2013-08-22

**Authors:** Mitsuaki Yanagida, Mikiko Kawasaki, Maki Fujishiro, Masako Miura, Keigo Ikeda, Kazuhisa Nozawa, Hiroshi Kaneko, Shinji Morimoto, Yoshinari Takasaki, Hideoki Ogawa, Kenji Takamori, Iwao Sekigawa

**Affiliations:** ^1^Institute for Environmental and Gender Specific Medicine, Juntendo University Graduate School of Medicine, 2-1-1 Tomioka, Urayasu-shi, Chiba 279-0021, Japan; ^2^Department of Internal Medicine, Juntendo University Urayasu Hospital, 2-1-1 Tomioka, Urayasu-shi, Chiba 279-0021, Japan; ^3^Department of Internal Medicine and Rheumatology, Juntendo University School of Medicine, 2-1-1 Hongo, Bunkyo-ku, Tokyo 113-8421, Japan; ^4^Department of Rheumatology, Kohnodai Hospital, National Center for Global Health and Medicine, 1-7-1 Kohnodai, Ichikawa-shi, Chiba 272-8516, Japan

## Abstract

Rheumatoid arthritis (RA) is a chronic inflammatory disorder of the synovial membrane that results in the destruction of bone and cartilage in affected joints. Tocilizumab is a biological agent and an anti-interleukin-6 (IL-6) receptor monoclonal antibody that blocks IL-6-mediated inflammatory processes in RA patients. In order to identify novel disease-related proteins and candidate biomarkers, we analyzed the changes in the serum proteome profiles of patients with RA who were treated with tocilizumab. Serum samples were collected from the RA patients before and after tocilizumab treatment. Following immunodepletion of major proteins, the proteins were digested and labeled with isobaric tag, iTRAQ reagent. The proteins were identified and quantified using liquid chromatography-tandem mass spectrometry. Among a total of 311 proteins identified, seven were decreased and 16 were increased by tocilizumab treatment. Although some of the proteins are known to be related to RA, several are currently unknown with respect to their relationship to RA and may be involved in the development of this disease. This study is the first to perform a comparative serum proteomic analysis of RA patients treated with tocilizumab. Our results may contribute to the identification of novel disease-related proteins and enhance the understanding of the pathogenesis of RA.

## 1. Introduction

Rheumatoid arthritis (RA) is a chronic inflammatory disorder of the synovial tissue that results in the destruction of joint cartilage and bone. RA is a multifactorial disease, and the precise molecular mechanisms underlying the pathogenesis of RA have not been fully elucidated. 

Recent progress has revealed that some inflammatory cytokines, such as tumor necrosis factor-*α* (TNF-*α*) and interleukin-6 (IL-6), contribute to the onset and progression of RA. Therefore, biological drugs targeting these specific molecules have been developed and are currently being applied in the treatment of RA. Several biological agents have been approved to treat RA, including infliximab (an anti-TNF-*α* mouse-human chimeric monoclonal antibody) [1], adalimumab (a fully human monoclonal antibody against TNF-*α*) [2], etanercept (a soluble TNF receptor-immunoglobulin chimeric protein) [3], tocilizumab (a humanized anti-interleukin-6 receptor monoclonal antibody) [4], and abatacept (a fusion protein of the extracellular domain of cytotoxic T-lymphocyte-associated antigen 4 and human immunoglobulin) [5]. These drugs effectively and rapidly improve the clinical condition of patients with RA in comparison to other traditional agents, such as disease-modifying antirheumatic drugs and anti-inflammatory drugs. Therefore, biological drugs cause drastic changes in the human body that are induced by targeted pathways and result in unexpected reactions. 

As drastic changes in the disease state are provoked by biological agents, differential analyses of proteins conducted before and after the administration of these drugs are an attractive and effective method of identifying disease-related proteins. RA is a multifactorial disease, and various biological pathways are related to its pathogenesis. Therefore, developing profiling methods based on multiple molecular biomarkers is expected to improve the diagnosis and prognosis of RA patients. Large-scale analytical methods, such as proteomic analyses, are suitable for identifying the set of proteins applied in the multiple biomarker method.

Previously, we conducted proteomic studies of the serum/plasma in RA patients treated with infliximab and etanercept using mass spectrometry and found that the detection levels of several proteins, including proteins related to the TNF-*α* and NF-kappa B responses, were changed by these treatments [6, 7]. Among them, the serum levels of two proteins, connective tissue growth factor (CTGF) and regenerating gene 1 alpha (REG1*α*), were confirmed to be increased in RA patients compared with those observed in healthy subjects. We also found that CTGF promotes osteoclast differentiation and activation, which can induce bone and joint destruction [8], and that REG1*α* activates synovial fibroblast cells, which promotes pannus progression [9]. Therefore, these large-scale approaches were useful for discovering novel disease-related proteins.

In this study, we investigated the serum proteome profiles of RA patients treated with tocilizumab. Tocilizumab is a fully humanized monoclonal antibody against the IL-6 receptor. It inhibits binding between IL-6 and the IL-6 receptor and blocks signal transduction through gp130. As this drug acts on RA via different pathways from those of anti-TNF-*α* agents, characteristic proteins must be identified using a differential proteomic analysis, which may result in the identification of novel disease-related proteins.

## 2. Materials and Methods

### 2.1. Materials

Sequencing-grade modified trypsin was obtained from Promega (Madison, WI, USA), and ammonium bicarbonate was obtained from Nacalai Tesque (Kyoto, Japan). HPLC-grade water, methanol, acetonitrile, dithiothreitol, iodoacetamide, and formic acid were purchased from Wako Pure Chemical Industries (Osaka, Japan). Multiple Affinity Removal System (MARS) Spin Cartridges Human-14 was purchased from Agilent (Santa Clara, CA, USA).

### 2.2. Patients and Samples

Before the start of the study, our local ethics committee, which conformed to the provisions of the World Medical Association's Declaration of Helsinki, approved the research protocol. Informed consent was obtained from all patients who participated anonymously in the study. The profiles of the RA patients are shown in Table 1. Seven female patients with RA (mean age: 68.3 years, age range: 51–93 years) were included in this study. All patients were diagnosed as having RA according to the criteria of the American College of Rheumatology, 1987 [10]. The duration of the disease ranged from 9 to 27 years. The Steinbrocker disease activity stage and functional class of each patient [11] are also described in Table 1. At the time of the start of tocilizumab treatment, all patients were being treated with prednisolone and/or methotrexate. Tocilizumab was administered once every four weeks. All patients exhibited a moderate or good response to tocilizumab based on the improvement rate calculated according to the disease activity score 28 (DAS28) [12].

Blood samples were obtained from each RA patient three times immediately prior to the first, second, and third administrations of tocilizumab. As a standard sample, we obtained blood from a healthy subject. All blood samples were incubated at room temperature for 30 minutes and centrifuged for 7 minutes at 3,000 rpm (1,600 g). The sera were stored at −80°C until the analysis.

### 2.3. Serum Pretreatment

The serum was pretreated with a MARS spin cartridge according to the manufacturer's instructions. Briefly, 8 *μ*L of the serum was diluted to 200 *μ*L with equilibration buffer and filtrated with ultrafiltration devices (Ultrafree, Millipore and Vivaspin500 MWCO = 300,000, Sartorius). The flow through fraction was applied to a MARS spin cartridge. The unbound fraction was recovered, and the protein concentration was measured according to the BCA method (BCA Protein Assay Reagent, Thermo Fisher Scientific, Waltham, MA, USA). The total of 25 *μ*g of protein in each fraction was used for the quantitative proteomic analysis. 

### 2.4. Trypsin Digestion and Stable Isotope Labeling

The protein samples were precipitated with ice-cold acetone and dissolved in 20 *μ*L of 0.5 mmol/L of triethylammonium bicarbonate buffer (pH 7.5). The following treatment was performed using the iTRAQ reagent kit according to the manufacturer's instructions. The samples were reduced with tris-(2-carboxyethyl) phosphine (TCEP) and alkylated with methylmethanethiosulfonate (MMTS). Trypsin was added to the samples, and the solutions were incubated at 37°C overnight. The peptide mixtures were then labeled with the iTRAQ 4plex reagent. The first, second, and third samples were labeled with iTRAQ115, iTRAQ116, and iTRAQ117 reagents, respectively. The sample of the standard serum was labeled with iTRAQ114 reagent. These four samples were mixed and purified with a strong cation exchange (SCX) column equilibrated with 10 mmol/L of potassium phosphate buffer in 25% acetonitrile (pH 3.0). The peptides were separated into 12 fractions using stepwise elution with 20, 30, 35, 40, 45, 50, 55, 75, 100, 150, 200, and 350 mmol/L potassium chloride. The peptide samples were desalted with a reversed-phase mini column cartridge (SepPak Light C18 cartridges, Waters, Milford, MA). Following evaporation, the peptides were reconstituted in 0.1% v/v formic acid.

### 2.5. Liquid Chromatography Coupled to Tandem Mass Spectrometry (LC-MS/MS) Analysis

The stable-isotope labeled peptides mixture was analyzed with a nanoflow LC-MS/MS using a direct nano-LC system (DiNa, KYA technologies, Tokyo, Japan) and a mass spectrometer (QSTAR-Elite, AB SCIEX, Framingham, MA, USA). Each fraction of the peptides obtained from the SCX column was injected into a C18 reversed-phase chromatography system (1 mm × 0.5 mm i.d. C18 trap column and 50 mm × 0.15 mm i.d. HiQ sil C18 separation column, KYA technologies, Tokyo, Japan) equilibrated with 0.1% formic acid and 2% acetonitrile. The peptides were eluted with a linear gradient of 2–35% acetonitrile with 0.1% v/v formic acid for 155 minutes. The flow rate was 200 nl/min.

The eluted peptides were introduced on-line into the mass spectrometer with a nanospray emitter (KYA technologies, Tokyo, Japan) and analyzed using one MS scan, followed by three information-dependent MS/MS acquisition scans of the three most intense MS peaks under a spray voltage of 1.8–2.0 kV.

### 2.6. Data Analysis

The twelve sets of acquired MS data were analyzed together using the ProteinPilot software package ver. 3.0 (AB SCIEX, Framingham, MA, USA.). The processed MS/MS peak list was compared with the RefSeq human protein database (2009. 05. 18; National Center for Biotechnology Information, Bethesda, MD, USA) containing 39,071 entries for human proteins. The search conditions were set to the experimental conditions, and the biological modifications set was considered. The criterion employed for protein identification was identification confidence (conf value) of ≥95. 

The relative protein content was quantified according to the area of the reporter MS/MS peak ion in the range of 114–117 m/z. The relative amount of each protein obtained from the RA patients was calculated as the ratio to the standard serum using the ProteinPilot software program, for example, Area115/Area114, Area116/Area114, and Area117/Area114. In order to compare the samples obtained before and four weeks after tocilizumab treatment (early phase), the ratio of Area116 to Area115 was calculated. The increase ratio was Area116/Area115, and the decrease ratio was Area115/Area116. In the comparison of the samples obtained before and eight weeks after tocilizumab treatment (late phase), the increase ratio was Area117/Area115 and the decrease ratio was Area115/Area117. The ratios of each protein obtained from the entire patient population were averaged.

The relative amounts of the identified proteins were also compared using a paired *t*-test. For the early phase, the values of Area115/Area114 and Area116/Area114 of each protein were compared, and for the late phase, the values of Area115/Area114 and Area117/Area114 of each protein were analyzed. The proteins for which the relative amounts changed by more than 1.5-fold at *P* < 0.05 in the paired *t*-test were defined as being changed by tocilizumab treatment.

## 3. Results

In this study, we investigated the influence of tocilizumab treatment on the serum proteome profiles of RA patients. As is widely known, the abundant quantity of proteins present in the serum at high concentrations, such as serum albumin and immunoglobulins, often obstructs the comprehensive proteomic analyses of serum [13, 14]. In order to overcome this obstacle, highly abundant proteins are usually depleted before conducting proteomic analyses. For this purpose, we used a multiple affinity removal system, which consisted of a column coupled with specific antibodies against the 14 most abundant proteins in the serum. The representative average yield of protein in these steps was 6.3%, and approximately 94% of the proteins were depleted. These values are typical, according to the manufacturer's instructions.

Table 1 shows the profiles of the seven RA patients who participated in this study. The DAS28 score of each patient decreased after tocilizumab treatment, indicating that tocilizumab therapy effectively improved the pathological condition of each patient.

The serum samples were treated with a MARS column, and an equal amount (25 *μ*g) of protein from the unbound fraction was used for a further quantitative analysis. The stable isotope-labeled peptide mixture was separated to 12 fractions; therefore, 12 LC-MS/MS analyses were performed per patient. The analyses were conducted for seven RA patients, and 311 proteins in total were significantly (conf value ≥95) identified in a ProteinPilot database search. The average fit global false discovery rate for the seven analyses was 0.52% (range: 0–1.8%).

According to our judging criteria described in the Materials and Methods section, we categorized the decreased proteins and increased proteins. The number of proteins whose increase ratio was more than 1.5-fold was 23 for the early phase and 31 for the late phase. The number of proteins whose decrease ratio was more than 1.5-fold was 15 for the early phase and 22 for the late phase. The number of changed proteins in the late phase was greater than that observed in the early phase. Judging from the change in the DAS28 score of each RA patient during the tocilizumab treatment, the protein profile in the late phase was more similar to that in the healthy state than in the early state.

We confirmed the results using paired *t*-tests (*P* < 0.05). Consequently, the expression levels of seven proteins were decreased by tocilizumab treatment in the late phase, of which three changed in both the early and late phases and four changed in the late phase only. A total of 16 proteins were increased by tocilizumab treatment, of which six changed in both the early and late phases and 10 changed in the late phase only. The proteins that exhibited significant changes from the early phase were considered to respond more rapidly to tocilizumab treatment. These differentially expressed proteins are shown in Tables 2 and 3.

## 4. Discussion

Among the differentially expressed proteins listed in Table 2, some are known to be related to RA. The serum levels of acute-phase proteins, such as orosomucoid 1 (*α*1-acid glycoprotein), C-reactive protein, haptoglobin, and the serine protease inhibitor clade A (*α*1-antitrypsin), were higher in the RA patients than in the normal controls, and tocilizumab treatment decreased the concentrations of these proteins. As these proteins are known to be directly induced by IL-6 [15] and upregulated under inflammatory conditions [16], the downregulation of these proteins resulted from the blockade of the IL-6 pathway by tocilizumab. These results showed that the analysis method used in this study is appropriate for detecting disease-related proteins.

Pregnancy zone protein (PZP) is a protein homologous to *α*2-macroglobulin [17]. It has been reported that the level of PZP is increased in the serum of RA patients [18]. Our results indicating that the level of PZP decreased following tocilizumab treatment are consistent with the findings of that report. However, it is unknown whether this protein is a direct target of the IL-6 pathway, and the functional relationship between the change in the protein level and the pathogenesis of RA also remains unexplained.

Recently, the serum concentration of leucine-rich *α*2-glycoprotein (LRG1) was reported to be elevated in RA patients and decreased by anti-TNF-*α* therapy [19]. In our study, LRG1 was decreased by tocilizumab treatment; therefore, it commonly responds to anti-TNF*α* and anti-IL-6 receptor drugs, although it is unclear whether LRG1 is directly induced by IL-6. This means that this protein may play an essential role in the etiology of RA. 

The proteins for which the serum levels increased are listed in Table 3. Among them, four apolipoproteins, apolipoproteins A-I, A-II, C-I, and C-II, were included. There are several reports regarding the levels of serum lipoproteins in RA patients treated with biological agents [20–22]. Ajeganova et al. reported that the levels of apolipoprotein A-I are slightly increased in the serum of RA patients treated with anti-TNF agents [20]. Kawashiri et al. reported that the serum levels of apolipoproteins A-I and A-II, HDL, and LDL in RA patients are significantly increased by tocilizumab treatment [21]. Conversely, Soubrier et al. reported that the levels of apolipoprotein A-I in the serum of RA patients are not significantly changed by anti-TNF-*α* therapy [22]. Our results demonstrated increased levels of apolipoproteins A-I and A-II and also C-I and C-II following tocilizumab treatment. Although our criteria for defining changed proteins in this analysis were not met, adiponectin also increased by more than 1.4-fold due to tocilizumab treatment (*P* < 0.05). Therefore, anti-IL-6 therapy influences the lipid metabolism in RA patients, as recently indicated [21]. A previous study reported that the tocilizumab treatment of patients with Castleman's disease increased the serum level of triglycerides, cholesterol, and their body mass index [23]. These phenomena could therefore be related to the increased production of apolipoproteins. 

Some other increased proteins listed in Table 3 are involved in the pathogenesis of RA. Retinol binding protein 4 is a carrier protein for retinol in the blood and was also increased by tocilizumab treatment in this study. The serum levels of vitamins A and E are known to be low in RA patients, which could be caused by the lower level of retinol binding protein [24]. Recently, it was reported that the serum level of this protein is increased by infliximab treatment [25]. Therefore, this protein may be commonly involved in the TNF-*α* pathway and IL-6 signal transduction.

Selectin-L (CD62) is a cell adhesion molecule that plays a regulatory role in inflammation [26]. In RA patients, the serum level of selectin L is lower than that observed in healthy controls [26]. Superoxide dismutase 3 is an antioxidant enzyme that protects tissues from oxidative stress. A previous report revealed the activity of this protein in RA patients to be lower than that observed in healthy subjects [27]. In our study, the levels of these two proteins increased following tocilizumab treatment, in accordance with previous reports.

Some reports have investigated the levels of proteins in the synovial tissue or fluid of RA patients. Lymphatic vessel endothelial hyaluronan receptor 1 (LYVE-1) is localized on the plasma membrane of endothelial cells in lymphatic vessels and is involved in the transport of hyaluronan [28]. One report demonstrated that lymphatic vessels are numerous in RA synovial tissues based on immunohistochemistry with anti-LYVE-1 [29]. However, the behavior of LYVE-1 in the serum of RA patients remains unknown. 

Melanoma cell adhesion molecule (MCAM/MUC18/CD146) is known to be a tumor progression marker of human melanoma [30] that plays a role in cell adhesion [31]. It has been reported that the levels of MCAM/MUC18/CD146 in the synovial fluid of RA patients are increased compared with those observed in healthy controls [32]. The authors reported that this finding may reflect the increased activity of endothelial cells and angiogenesis in the synovial tissues of RA patients [32]. In this study, the serum level of MCAM/MUC18/CD146 increased following tocilizumab treatment. Therefore, the concentration of this protein in the serum does not vary in direct proportion to that in the synovia.

The expression levels of other proteins related to the sex hormone function were increased by tocilizumab treatment. Sex hormone-binding globulin (SHBG) is a carrier protein for sex hormones, such as androgens and estrogens, in the plasma [33]. It has been reported that the serum levels of SHBG in RA patients are lower than those observed in control subjects [34] and that TNF-*α* downregulates the expression of SHBG [35]. The increase in the level of SHBG in the serum following tocilizumab treatment observed in this study may have been induced by IL-6 receptor signaling or consequent TNF-*α* suppression.

Cysteine-rich secretory protein 3 (CRISP-3) is an androgen-regulated protein [36] that is upregulated in prostate carcinomas [37]. It has been reported that the serum levels of androgens, such as testosterone and dehydroepiandrosterone sulfate, in RA patients are decreased compared with those observed in healthy subjects [38]. Therefore, CRISP-3 may also be suppressed in RA patients. Our results showing that the level of CRISP-3 was increased by tocilizumab treatment are consistent with this concept.

Despite these favorable findings, the methods used for a large-scale analysis of serum/plasma proteome have some limitations in detecting whole proteins. One of the reasons for this is that some of the proteins are removed during the process of protein purification. For example, fibrinogen, which is known to be downregulated by tocilizumab treatment [23], is consumed and removed from the sample by clotting during the serum preparation. Therefore, we could not detect the decrease of fibrinogen. Another reason is that mass spectrometry does not have a high dynamic range or sufficient sensitivity to detect the whole serum proteins, which are present in a wide range of concentrations. Low abundance proteins, such as cytokines, including IL-6 and TNF-*α*, are out of the detection range. Therefore, the establishment of more comprehensive methods for serum/plasma proteome analysis is highly anticipated.

Thus, the methods for serum/plasma proteomics are still developing. However, the changed proteins mentioned above possibly play a role in the pathogenesis of RA, although their involvement in RA has not been experimentally elucidated. The relationships between the other increased proteins listed in Table 3 and RA also remain unknown; therefore, these proteins may be novel candidate RA disease-related proteins. 

## 5. Conclusions

We and other groups have conducted comparative proteomic studies of RA patients treated with biological agents [6, 7, 39, 40]. These studies have discovered several interesting proteins related to the pathogenesis of RA [8, 9, 41]. In order to further identify proteins implicated in the etiology of RA, we performed a mass spectrometry-based comparative proteomic analysis of the serum of RA patients treated with tocilizumab. 

In this study, we succeeded for the first time in detecting several proteins that were differentially expressed in serum samples obtained before and after tocilizumab treatment. Generally, large-scale mass spectrometry-based proteomic analyses of clinical samples obtained from patients tend to attach greater importance to comprehensiveness in identifying protein sets than to the reliability of the quantitative data for an individual protein. Therefore, in order to demonstrate the relationship between each protein and the pathogenesis of RA, we will conduct a future validation study of these proteins in a larger number of patients using more specific quantitation methods and functional assays. Further investigations of these proteins could therefore help to discover novel biomarker proteins related to the etiology of RA. 

## Figures and Tables

**Table 1:** Profiles of the patients with RA.

No.	Age (years)	Sex	Duration of RA (years)	Class/stage^†^	DAS28-ESR			PSL (mg/day)	MTX (mg/week)
					0	4W	8W		
1	57	F	18	2/4	5.01	3.16	2.93	2.5	6
2	93	F	11	2/4	5.32	3.52	3.00	3	6
3	91	F	20	1/2	3.65	2.38	2.66	2	6
4	51	F	—	2/3	5.82	2.13	2.06	5	5
5	55	F	21	2/4	4.62	2.66	3.27	0	2
6	68	F	9	1/4	4.05	4.05	1.66	4	0
7	63	F	27	3/4	4.93	3.63	2.98	5	4

†: Functional (class) and radiological (stage) classifications by Steinbrocker et al. [11].

DAS28: disease activity score 28 [12]. The DAS28 was evaluated at the time of serum collection. 0: before the first treatment, 4W: before the 2nd treatment, 8W: before the 3rd treatment.

PSL: dose of prednisolone, MTX: dose of methotrexate.

**Table 2:** Proteins identified to decrease after tocilizumab treatment.

Protein name	Accession no.	Times of detection	Relative abundance (average)				Decrease ratio		Paired *t*-test *P *	
			ctr	Before	4W	8W	Before/4W	Before/8W	Before versus 4W	Before versus 8W
C-reactive protein, pentraxin-related	55770842	5	1	13.507	4.107	2.124	**17.928**	**15.085**	0.252	**0.037**
Orosomucoid 1 precursor	167857790	6	1	6.130	1.725	0.775	**13.070**	**7.750**	0.124	**0.041**
Haptoglobin isoform 1 preproprotein	4826762	7	1	2.876	1.028	0.507	**7.261**	**5.266**	0.067	**0.015**
Serine proteinase inhibitor, clade A, member 1	50363221	7	1	3.278	1.403	1.025	**3.974**	**3.318**	0.051	**0.008**
Pregnancy-zone protein	162809334	7	1	1.592	0.755	0.633	**2.126**	**2.966**	**0.010**	**0.009**
Complement component 9 precursor	4502511	7	1	1.979	1.194	1.307	**1.713**	**1.631**	**0.012**	**0.015**
Leucine-rich alpha-2-glycoprotein 1	16418467	7	1	2.636	1.681	1.735	**1.803**	**1.597**	**0.012**	**0.013**

Accession number: accession number (gi) in the human RefSeq protein database of the National Center for Biotechnology Information, USA.

Times of detection: the number of samples in which the protein was detected.

Relative abundance: the ratio of the iTRAQ reporter ion to the 114 reporter ion (control).

Decrease ratio: the ratio of protein abundance between the first and seconed samples (Before/4W) and between the first and third samples (Before/8W).

Bold: increased by more than 1.5-fold.

Paired *t*-test *P*: the *P* value of a paired *t*-test between the first and second samples (4W) or between the first and third samples (8W).

Bold: *P* < 0.05.

**Table 3:** Proteins identified to increase after tocilizumab treatment.

Protein name	Accession no.	Times of detection	Relative abundance (average)				Increase ratio		Paired *t*-test *P *	
			ctr	Before	4W	8W	4W/before	8W/before	Before versus 4W	Before versus 8W
Apolipoprotein A-I preproprotein	4557321	7	1	0.942	1.632	1.529	**1.502**	**1.515**	0.111	**0.040**
Superoxide dismutase 3, extracellular precursor	118582275	7	1	1.218	1.672	1.806	1.442	**1.509**	**0.046**	**0.046**
Sex hormone-binding globulin isoform 1 precursor	7382460	7	1	1.496	2.061	2.337	1.376	**1.510**	**0.013**	**0.027**
Lymphatic vessel endothelial hyaluronan receptor 1	40549451	5	1	1.299	1.713	1.994	1.340	**1.505**	**0.028**	**0.037**
ABI gene family, member 3 (NESH) binding protein	33667044	5	1	1.143	1.629	1.875	1.403	**1.603**	**0.036**	**0.049**
Apolipoprotein C-II precursor	32130518	7	1	1.092	1.777	1.811	**1.637**	**1.591**	**0.044**	**0.032**
Chitobiase, di-N-acetyl-	4758092	5	1	0.793	1.062	1.219	1.350	**1.564**	**0.020**	**0.011**
Lysosomal-associated membrane protein 1	112380628	5	1	1.417	1.648	2.239	1.220	**1.581**	**0.017**	**0.037**
Selectin L precursor	4506875	5	1	1.022	1.389	1.753	1.371	**1.674**	**0.006**	**0.035**
Retinol-binding protein 4, plasma precursor	55743122	7	1	1.150	1.797	1.920	**1.607**	**1.637**	**0.016**	**0.041**
Cysteine-rich secretory protein 3	5174675	7	1	0.799	1.224	1.325	**1.529**	**1.685**	**0.002**	**0.004**
Melanoma cell adhesion molecule	71274107	7	1	1.265	1.758	2.153	1.375	**1.705**	**0.009**	**0.000**
Afamin precursor	4501987	7	1	0.962	1.491	1.676	**1.574**	**1.733**	**0.004**	**0.010**
Apolipoprotein A-II preproprotein	4502149	7	1	0.770	1.433	1.363	**1.822**	**1.745**	**0.017**	**0.008**
Apolipoprotein C-I precursor	4502157	5	1	1.008	1.880	1.975	**1.869**	**1.896**	**0.010**	**0.039**
Fumarylacetoacetate hydrolase	4557587	4	1	1.022	1.836	1.966	**1.965**	**2.087**	0.155	**0.038**

Accession number: accession number (gi) in the human RefSeq protein database of the National Center for Biotechnology Information, USA.

Times of detection: the number of samples in which the protein was detected.

Relative abundance: the ratio of the iTRAQ reporter ion to the 114 reporter ion (control).

Increase ratio: the ratio of protein abundance between the second and first samples (4W/before) and between the third and first samples (8W/before).

Bold: increased by more than 1.5-fold.

Paired *t*-test *P*: the *P* value of a paired *t*-test between the first and second samples (4W) or between the first and third samples (8W).

Bold: *P* < 0.05.
